# Ultrasonography-Guided Fine Needle Aspiration Cytology Versus Histopathology: Diagnostic Consistency in Salivary Gland Neoplasms

**DOI:** 10.7759/cureus.69552

**Published:** 2024-09-16

**Authors:** Ajit Kumar, Jeeshan Khan, Gursimrat Kaur, Mohammed Khalilullah, Asok K Saha, Alok Kumar

**Affiliations:** 1 Otolaryngology - Head and Neck Surgery, All India Institute of Medical Sciences (AIIMS) Deoghar, Deoghar, IND; 2 Otolaryngology - Head and Neck Surgery, Government District Hospital Kuchaman City, Kuchaman City, IND; 3 Otolaryngology - Head and Neck Surgery, Mata Gujri Memorial Medical College and Lions Seva Kendra (LSK) Hospital, Kishanganj, IND; 4 Otolaryngology, Kolkata Medical College and Hospital, Kolkata, IND; 5 Community and Family Medicine, All India Institute of Medical Sciences (AIIMS) Deoghar, Deoghar, IND

**Keywords:** cytohistological correlation, fine needle aspiration cytology (fnac), parotid gland tumor, pleomorphic adenoma, salivary gland tumor

## Abstract

Introduction

Salivary gland lesions include a diverse range of histological types and biological behaviors, making accurate diagnosis and effective treatment challenging for specialists. Fine needle aspiration cytology (FNAC) plays a significant role in making preoperative diagnoses and further plans of surgery. In differentiating malignant from benign salivary gland tumors, the diagnostic accuracy of fine needle aspiration cytology (FNAC) is high. However, the final diagnosis is histopathological. The aim of this study is to correlate cytological and histopathological interpretations in salivary gland lesions.

Materials and method

Thirty patients were included in this study, all of them were diagnosed with salivary gland swellings and operated on in the ear, nose, and throat (ENT) department of Mata Gujri Memorial (MGM) Medical College, Kishanganj, in the northeastern region of India, between December 2020 and November 2022. Ultrasonography (USG) and computed tomography (CT) scans were performed in each case, and FNAC was done to make a preoperative diagnosis. After surgery, specimens were collected for histopathological examination. A correlation was then made between the findings of the preoperative cytological examination and the results of the postoperative histopathological examination. The comparison was performed using a 2×2 table, and the analysis of sensitivity, specificity, positive predictive value (PPV), and negative predictive value (NPV) was carried out using Microsoft Excel (Microsoft Corp., Redmond, WA) and SPSS software (IBM SPSS Statistics, Armonk, NY).

Results

An equal number of male and female samples were included in the study, in which parotid involvement was predominant (60%). On FNAC, 53% of parotid gland tumors were found to be benign, whereas only 23.3% of submandibular gland tumors were benign. Pleomorphic adenoma was the most common finding. In the present study, the sensitivity, specificity, positive predictive value, and negative predictive value of USG-guided FNAC, compared to the gold standard histopathology, were 96%, 33.3%, 92.3%, and 50%, respectively. The diagnostic accuracy was found to be 89.3%.

Conclusion

The findings of this study suggest that FNAC is a reliable technique for the preoperative diagnosis of salivary gland tumors, as it is minimally invasive and offers valuable diagnostic information. An accurate cytological diagnosis can help avoid unnecessary surgery.

## Introduction

In India, salivary gland tumors are a significant concern, with many individuals seeking medical attention upon noticing the swelling. Salivary gland tumors account for about 3%-10% of all neoplasms in the head and neck region [1]. The nature of salivary gland swelling can vary, ranging from non-neoplastic to neoplastic. Approximately 21.7% of all salivary gland neoplasms are malignant [2,3]. Three-fourths of parotid gland tumors are benign, while the majority of minor salivary gland tumors are malignant [4]. Ear, nose, and throat (ENT) surgeons find surgeries for salivary gland tumors particularly compelling. The decision to perform surgery relies heavily on accurate and efficient diagnostic techniques.

In both Europe and Asia, ultrasonography (USG) is considered the primary imaging technique for evaluating salivary glands, lymph nodes, and other soft tissue pathologies in the head and neck region [5]. In certain cases, ultrasonography (USG) may be insufficient for the complete visualization and analysis of lesions due to their anatomical location, such as the deep lobe of the parotid gland or the region posterior to the mandible. In these instances, it is strongly advised to perform additional imaging investigations, such as computed tomography (CT) or magnetic resonance imaging (MRI), for a more comprehensive evaluation [6].

Fine needle aspiration cytology (FNAC) is a cytological method based on morphological findings of individual and small groups of cells aspirated using a fine needle. FNAC was introduced in the 1920s, and soon, it gained wide acceptance among clinicians due to the ease of its performance and rapidity of diagnosis [7]. For over 30 years, FNAC has been utilized to diagnose lesions of the salivary glands. This procedure will avoid incisional biopsy and the risks of fistula formation or tumor implantation in case of neoplasms [8]. It has been proven effective in both diagnosing these lesions and distinguishing between neoplastic and non-neoplastic diseases [9].

Additionally, FNAC can be guided with the help of CT and ultrasound, improving the precision of cytological diagnosis. However, histopathology remains the most accurate diagnostic method, as many benign and malignant tumors exhibit overlapping cytological features, making accurate diagnosis challenging [10]. By comparing cytological findings with histopathological results, this study aims to determine the reliability and efficacy of USG-guided FNAC in diagnosing salivary gland neoplasms. The objective of this study is to assess the most common age group and the most commonly found tumor during the study period. This study also evaluates the diagnostic accuracy of USG-guided FNAC in detecting salivary gland tumors by analyzing the correlation between USG-guided FNAC results and histopathological findings. The comparison is made by assessing the sensitivity, specificity, negative predictive value (NPV), and positive predictive value (PPV) of USG-guided FNAC in relation to histopathology.

## Materials and methods

This research employed a prospective observational design. It was carried out from December 2020 to November 2022 at the Mata Gujri Memorial (MGM) Medical College's Department of Otorhinolaryngology-Head Neck Surgery in Kishanganj, Bihar. The following formula was used to obtain the sample size: n = 2×SD2×(Za/2+Zb)2/d2, where n is the required sample size, SD is the standard deviation, Za/2 is Z0.05/2 = Z0.025 = 1.96 at type 1 error of 5%, Zb is Z0.20 = 0.842 at 80% power, and d is the effect size, which is the difference between mean values. After substituting the values, the sample size was determined to be 30.

**Figure 1:**
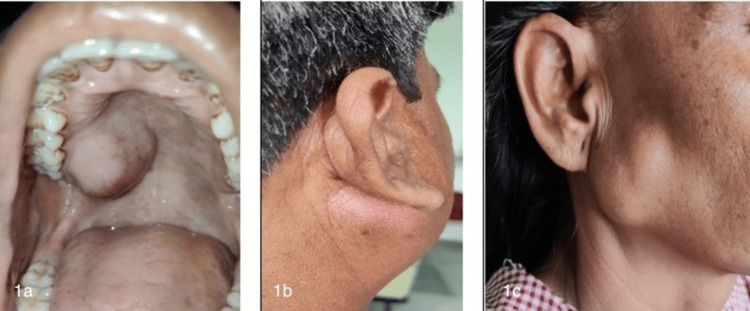
Salivary gland swellings 1a: Minor salivary gland swelling over the palate. 1b: Retro-auricular fullness (a sign of parotid swelling). 1c: Typical bosselated surface of pleomorphic adenoma.

Therefore, a total of 30 patients, aged 15-65 years, who were diagnosed with salivary gland neoplasm were included in the study. Appropriate institutional ethics clearance from the Ethical Committee of Mata Gujri Memorial (MGM) Medical College and Lions Seva Kendra (LSK) Hospital, Kishanganj (approval number: MGM/IEC-72/2023) and informed consent were obtained. The study excluded patients with neoplasms involving the skin, those below 15 or above 65 years of age, and individuals with comorbidities such as uncontrolled diabetes, resistant hypertension, chronic obstructive pulmonary disease (COPD), and cardiovascular or neurological disorders. Clinical details and findings from local examinations were recorded (Figure 1).

**Figure 2:**
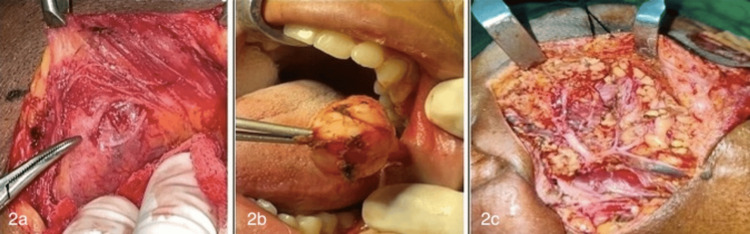
Intraoperative pictures 2a: Marginal mandibular nerve. 2b: Excision of minor salivary gland tumor. 2c: Pes anserinus (facial nerve).

Ultrasonography evaluated the size, shape, extent, and involvement of structures. A 23 G needle was used for FNAC, and May-Grunwald-Giemsa stain was applied to the aspirate. After receiving a cytological diagnosis, the patients had the necessary surgical procedures performed (Figure 2), and the specimens were examined by histopathological method. Post-surgery, patients were followed up at 7-10 days and subsequently at three, six, and 12 weeks.

The gold standard for determining the sensitivity and specificity of FNAC was the histological diagnosis. To perform cytohistological correlation, relevant statistical tests were run. Factors such as age, gender, lesion site, and cytological and histological features were all analyzed in these instances.

After the data was put into a Microsoft Excel spreadsheet (Microsoft Corp., Redmond, WA), SPSS version 27.0 (IBM SPSS Statistics, Armonk, NY) was used for analysis. Numerical variables were summarized using means and standard deviations (SD), while categorical variables were summarized using counts and percentages. Significant differences in proportions were evaluated using the chi-square test. A p-value of less than 0.05 was deemed statistically significant.

## Results

In our study, we had 15 individuals of each gender, comprising males and females. Table 1 shows that the majority of the patients were in the 15-30 age range. The p-value was 0.0048, suggesting statistical significance.

**Table 1:** Distribution of age groups

Age groups (years)	Numbers
15-30	14
31-44	4
45-65	12

In our study, the most commonly involved gland was the parotid (60% of cases). Submandibular gland involvement was observed in 10 patients, while minor salivary gland involvement was observed in only two patients. The z-value calculated was 4.3818, with a p-value of <0.00001, indicating significant findings at p<0.05.

On USG-guided FNAC, 80% of cases were found to be benign, 6.6% as malignant, and 13% as non-neoplastic (Table 2).

**Table 2:** Results of USG-guided FNAC USG: ultrasonography, FNAC: fine needle aspiration cytology

Glands	Benign	Malignant	Non-neoplastic
Parotid	15	1	2
Submandibular	7	1	2
Minor salivary gland	2	0	0

After undergoing the necessary surgical procedures, specimens were sent for histopathological analysis. The results showed that 25 (83.3%) cases were benign, three (10%) cases were malignant, and two (6.6%) cases were non-neoplastic.

**Figure 3:**
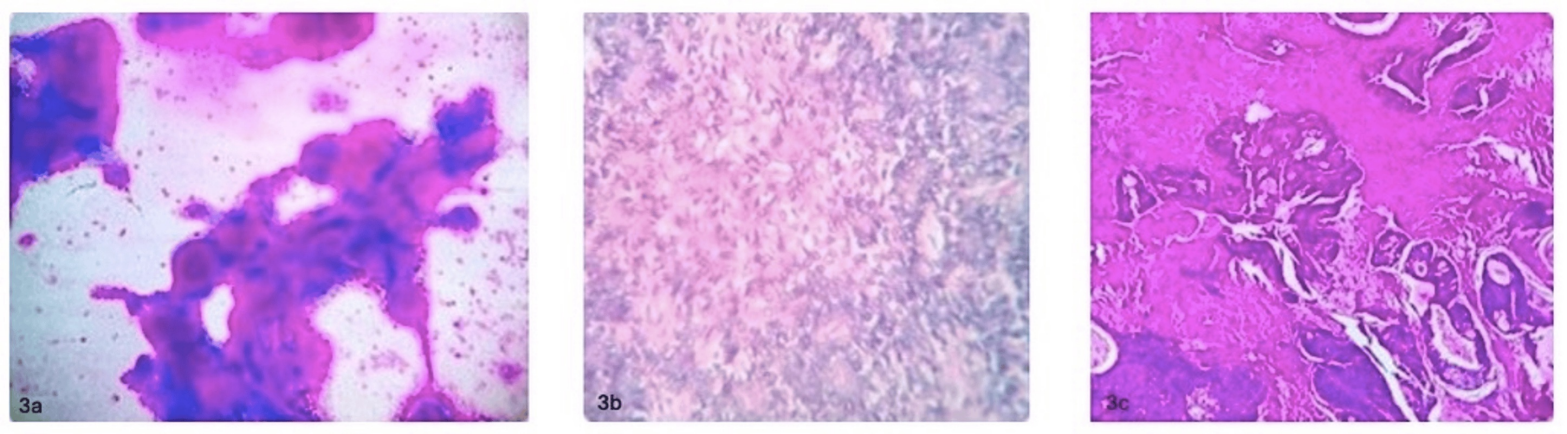
Cytological and histopathological slides of salivary gland tumors (pictures were taken from 48MP Sony IMX586 mobile camera through microscope eyepiece) 3a: FNAC: pleomorphic adenoma. 3b: Histopathology: pleomorphic adenoma. 3c: Histopathology: mucoepidermoid carcinoma. FNAC: fine needle aspiration cytology

In our study, pleomorphic adenoma (Figure 3a, 3b) was the most frequent benign histopathological finding, observed in 18 cases. Mucoepidermoid carcinoma (Figure 3c), identified in two cases, was the most prevalent malignant tumor (Table 3).

**Table 3:** Results on histopathology

Benign tumor distribution	Number
Pleomorphic	18
Myoepithelial	1
Basal cell adenoma	1
Angio-lymphoid hyperplasia	1
Oncocytoma	1
Benign lymphoepithelial cyst	2
Warthin's tumor	1
Malignant tumor distribution	Number
Myoepithelial carcinoma	1
Mucoepidermoid carcinoma	2

A p-value of 0.0009 and a chi-square value of 10.9959 indicated statistical significance in the relationship between USG-guided FNAC and histology. The sensitivity and specificity of FNAC were 96% and 33.3%, respectively. Table 4 shows that the diagnostic accuracy was 89.3% overall, with a positive predictive value of 92.3% and a negative predictive value of 50%.

**Table 4:** Association between USG-guided FNAC versus histopathology USG: ultrasonography, FNAC: fine needle aspiration cytology

	Histopathology		Total
FNAC	Benign	Malignant	
Benign	24	2	26
Malignant	1	1	2
Total	25	3	28

## Discussion

There were 30 patients in total in this study, with a similar distribution of male and female participants. Regardless of gender, the majority of participants (46.6%) were between the ages of 15 and 30 years old, with a mean age of 35 years. In a related study, Vedula et al. included 68 specimens of salivary gland tumors, in which 58.82% of cases were females compared to males (41.18%) [11].

In comparison to other salivary glands, the parotid gland had the highest number of tumor cases (n=18) when it came to the distribution of salivary gland tumors. Similar results were found in a multicentric investigation on the distribution and frequency of salivary gland tumors conducted by Alsanie et al. [12].

A comprehensive preoperative workup including cytological and radiographic examinations is necessary when planning for salivary gland surgery. Because fine needle aspiration cytology (FNAC) samples only a small region, it can occasionally result in inadequate sampling and diagnostic mistakes. Notwithstanding this drawback, FNAC is useful in lesion diagnosis and histopathology correlation. It aids in determining the degree of neoplastic cell differentiation, which helps direct surgical intervention decisions.

The scope of surgery is determined by classifying malignant lesions as low or high grade, taking into account factors including the need to dissect the neck in cases of malignant parotid gland tumors and the preservation of the facial nerve. Tumor size and location affect the likelihood of malignancy; for example, 20%-25% of tumors in the parotid glands are malignant. More than 90% of tumors in the sublingual gland are malignant, and this figure rises to 40% for tumors in the submandibular glands [13].

In our study, benign tumors of the parotid gland (53.3%) outnumbered malignant lesions (6.6%), with pleomorphic adenoma being the most common pathology. In the submandibular gland, benign tumors were present in 23.3% of cases and malignant tumors in 3.33% of cases. Benign tumors (6.6%) were most frequently found in minor salivary glands. In the study by Gudmundsson et al., benign and malignant parotid gland tumors accounted for 90.3% and 9.6% of cases, respectively. Pleomorphic adenoma was the most frequent tumor, accounting for 63% of cases, followed by Warthin's tumor (17.5%) [14].

Upon correlating USG-guided FNAC findings with histopathology, we observed that two cases initially diagnosed as non-neoplastic on FNAC were later confirmed as benign neoplasms on histopathology. Conversely, one case diagnosed as benign on FNAC turned out to be malignant upon histopathological examination. Therefore, in the present study, the sensitivity was found to be 96% and specificity was 33.3%. According to this study, the negative predictive value was 50% and the positive predictive value was 92.3%. The accuracy of the diagnosis was 89.3%. The results of a related investigation by AlGhamdi et al. showed that the specificity was 100% and the sensitivity was 90.3%. Furthermore, 100% was reported as the matching positive predictive value and 57.1% as the corresponding negative predictive value. The accuracy of the diagnosis was 91.4% [15].

Limitation of the study

The small sample size limits the reliability of the study, and without randomization, there is a higher chance of bias affecting the results.

## Conclusions

The preoperative diagnosis of tumors is crucial for early detection and timely treatment, as it allows adequate and essential time for intervention. In this study, when performed by skilled pathologists, USG-guided FNAC proves to be a minimally invasive and reliable technique for diagnosing salivary gland tumors preoperatively, with sensitivity and specificity values of 96% and 33.3%, respectively. Its accuracy can prevent unnecessary procedures and provide essential information for future treatment planning. However, despite these advantages, overlapping cytological features may result in occasional diagnostic errors. In such cases, histopathology remains the gold standard for definitive diagnosis.

## References

[1] (2005). Pathology and genetics of head and neck tumours. WHO Classification of Tumours, 3rd Edition.

[2] Guzzo M, Locati LD, Prott FJ, Gatta G, McGurk M, Licitra L (2010). Major and minor salivary gland tumors. Crit Rev Oncol Hematol.

[3] Geiger JL, Ismaila N, Beadle B (2021). Management of salivary gland malignancy: ASCO guideline. J Clin Oncol.

[4] S Aruna, Pai P, Kittur SK (2016). Cytomorphological study of major salivary gland lesions: a 5-year experience at a tertiary care center. Med Inn.

[5] Alyas F, Lewis K, Williams M, Moody AB, Wong KT, Ahuja AT, Howlett DC (2005). Diseases of the submandibular gland as demonstrated using high resolution ultrasound. Br J Radiol.

[6] Kamble RC, Joshi AN, Mestry PJ (2013). Ultrasound characterization of salivary lesions. Int J Otorhinolaryngol Clin.

[7] Dudheon LS, Patrick CV (1927). A new method for the rapid microscopical diagnosis of tumors: with an account of 200 cases so examined. Br J Surg.

[8] Chatterjee T, Panda PK (2000). A pathological study of benign and malignant tumours of salivary glands. Med J Armed Forces India.

[9] Dharwadkar A, Paul B, Buch A, Agarwal N, Naik M, Gore C (2022). Cytological study of salivary gland lesions along with histopathological correlation in a tertiary care centre. Int J Sci Res Dent Med Sci.

[10] Sonal V (2016). Fine needle aspiration cytology of salivary gland lesions: study in a tertiary care hospital of North Bihar. Int J Res Med Sci.

[11] Vedula B, Srikanth Reddy K, Sree Ramulu Naidu R, Sudhakar R (2020). Histopathological study of salivary gland tumours. J Evid Based Med Healthc.

[12] Alsanie I, Rajab S, Cottom H (2022). Distribution and frequency of salivary gland tumours: an international multicenter study. Head Neck Pathol.

[13] Loyola AM, de Araújo VC, de Sousa SO, de Araújo NS (1995). Minor salivary gland tumours. A retrospective study of 164 cases in a Brazilian population. Eur J Cancer B Oral Oncol.

[14] Gudmundsson JK, Ajan A, Abtahi J (2016). The accuracy of fine-needle aspiration cytology for diagnosis of parotid gland masses: a clinicopathological study of 114 patients. J Appl Oral Sci.

[15] AlGhamdi GZ, Alzahrani AK, Saati H (2021). Correlation between fine needle aspiration cytology (FNAC) and permanent histopathology results in salivary gland masses. Cureus.

